# Transforming post pandemic cancer services

**DOI:** 10.1038/s41416-024-02596-9

**Published:** 2024-03-15

**Authors:** Thomas Round, Lakshman Sethuraman, Mark Ashworth, Arnie Purushotham

**Affiliations:** 1 School of Life Course and Population Sciences, King’s College London, London, UK; 2 Karkinos Healthcare Private Limited, Mumbai, India; 3 School of Cancer and Pharmaceutical Sciences, King’s College London, London, UK

**Keywords:** Health care, Diagnosis

## Abstract

This paper outlines the impact of the COVID-19 pandemic on cancer services in the UK including screening, symptomatic diagnosis, treatment pathways and projections on clinical outcomes as a result of these care disruptions. A restoration of cancer services to pre-pandemic levels is not likely to mitigate this adverse impact, particularly with an ageing population and increased cancer burden. New cancer cases are projected to rise to over 500,000 per year by 2035, with over 4 million people living with and beyond cancer. This paper calls for a strategic transformation to prioritise effort on the basis of available datasets and evidence—in particular, to prioritise cancers where an earlier diagnosis is feasible and clinically useful with a focus on mortality benefit by preventing emergency presentations by harnessing data and analytics. This could be delivered by a focus on underperforming groups/areas to try and reduce inequity, linking near real-time datasets with clinical decision support systems at the primary and secondary care levels, promoting the use of novel technologies to improve patient uptake of services, screening and diagnosis, and finally, upskilling and cross-skilling healthcare workers to expand supply of diagnostic and screening services.

## Cancer services and the pandemic

Coronavirus disease 2019 (COVID-19) impacted on all aspects of cancer diagnosis and care, including screening, diagnostic and treatment pathways. In this article we consider the evidence of impact on patients, healthcare professionals across primary and secondary care and the wider healthcare system, with proposed potential opportunities for the transformation of services.

By late 2021, more than 47,000 people were “missing” a cancer diagnosis in the UK [1], with an estimate for National Health Service (NHS) in England to work at 110% of previous capacity for 17 months to catch up on missing cancer diagnoses.

### Impact on the health service

#### Screening

All three national cancer screening programmes were significantly impacted with breast cancer screening being worst hit [2] with a decline of over 15% in the first year of the pandemic. The observed reductions in screening performance may be explained by the temporary suspension of national screening programmes during the early stages of the pandemic in England, and the inability or unwillingness of invitees to partake in screening once the programmes had been restored. As such, increased capacity to deliver additional screening appointments is needed to work through the backlog of outstanding patients who are eligible to attend.

#### Health service activity

Prior to the start of the pandemic, primary care urgent suspected cancer (USC) (or 2-week wait (2WW)) referrals increased to over 2 million per year [3], with a subsequent increase in cancer detection, decrease in emergency presentations (EPs) and improved patient outcomes [4].

During the pandemic lockdowns, there were significant decreases in non-COVID health service activity, including presentation to emergency departments and primary care. GP consultations for potential cancer clinical features decreased by 24% between 2019 and 2020 [5], particularly in the 6–12 weeks following the first national lockdown. There were approximately 300,000 fewer USC referrals in 2020/21 compared to 2019/20. Despite the drop in referrals the detection rate continued to improve to 54.8% (145,495 cancers) picked up following USC referral [3, 6].

The UK has one of the lowest diagnostic capacities in the developed world (computed tomography (CT), magnetic resonance imaging, endoscopy) [7] and the pandemic has had a direct impact on already stretched services. Rutter et al. [8] analysed diagnostic procedures and found that endoscopy activity reduced to 12% of pre-COVID levels in March–May 2020.

There has been a transformation in health services during the pandemic and post-pandemic, including the use of telephone, video and digital access, with strengths and weaknesses, such as potential digital access barriers for older and more underserved patients [9].

#### Waiting times

Analysis of NHS backlog data [10] found that prior to the pandemic there were already 4.43 million people on a waiting list, with more recent data showing a record of over 7 million people waiting for treatment in May 2023. The combination of ongoing pressure on services, the backlog of care and chronic workforce shortages means that all waiting times have increased to record high levels. Despite improvements, the number of elective and outpatient attendances currently being carried out is only now reaching pre-pandemic levels, while primary care activity is exceeding pre-pandemic levels [10].

#### Staff burnout

The European Society for Medical Oncology Resilience Task Force surveys [11, 12] found that the proportion of respondents reporting feelings of burnout was 49%, and the proportion at risk of distress had increased from 25% to 33% between the 2 surveys conducted in April 2020 and July 2020, respectively. Compared with the initial period of the pandemic, more participants reported feeling overwhelmed with workload and 25% were considering changing their future career with 38% contemplating leaving the profession.

A report published by the House of Commons Health and Social Care committee [13] found that “Trust leaders were concerned about staff well-being, stress and burnout” and “highlighted that nearly half of the doctors surveyed reported suffering from depression, anxiety, stress, burnout, emotional distress or another mental health condition”.

### Impact on patient outcomes

Maringe et al. [14] estimated the impact of diagnostic delays over a 12-month period from March 2020 up to 1, 3, and 5 years after diagnosis. Across four major tumour types, breast, colorectal, lung, and oesophageal, 3291–3621 avoidable deaths and an additional 59,204–63,229 life years lost could be attributable to delays in cancer diagnosis as a result of the COVID-19 lockdown in the UK.

Sud et al. [15] called for a prioritised approach in designing the recovery for “patient groups for whom delay would result in most life-years lost warrants consideration as an option for mitigating the aggregate burden of mortality in patients with cancer”.

Purushotham et al. [16] examined the stage of diagnosis of patients with cancer presenting during the pandemic compared with pre-pandemic. There was an overall 3.9% increase in advanced stage presentation (stages 3 and 4). The greatest shifts were seen in lung (absolute increase of 6.3%) and colorectal (5.4%) cancers. Similar studies exist for other cancers [17, 18].

The longer term impact of the pandemic across primary and secondary care is still being debated, and clearly outlines the need for both renewal and transformation of cancer services.

## Transforming cancer services

It is important to consider the whole cancer diagnosis pathway when considering opportunities for earlier detection and diagnosis [19]. Even before the pandemic, with an ageing population cancer incidence is projected to rise to over 500,000 new diagnoses per year by 2035, with over 4 million people living with and beyond cancer, placing significant strain on all aspects of healthcare provision. Pre-existing health inequalities have been exacerbated by the pandemic. In addition to resuming and sustaining regular cancer services and mitigating any adverse impacts on patient outcomes, the circumstances call for a transformation in cancer service delivery.

### A prioritised approach to post pandemic services

Services recovery with no prioritisation could lead to adverse outcomes and potentially exacerbate pre-existing inequalities. The approach recommended in this paper suggests prioritisation in a sequence of steps—prioritise cancers where an earlier diagnosis is feasible and clinically useful, focus on mortality benefit including by reducing EPs using data and analytics, focus on variation to try and reduce inequalities, with the potential for novel approaches across primary and secondary care.

Routes to diagnosis (RTD) [20] defines a methodology and dataset by which the route the patient follows to the point of diagnosis can be categorised. This paper discusses using RTD data to prioritise areas of focus and develop an optimised strategy to improve outcomes for cancer patients.

Patients diagnosed following an EP are known to experience poorer outcomes than other RTD, such as a general practitioner (GP) referral or screen-detected cancer (Table 1) [20]. Increasing primary care referrals leading to cancer diagnoses has been shown to improve outcomes—including mortality reductions with a significant proportion explained by earlier stage diagnosis [4]. Reducing EPs has the potential to improve overall cancer survival, reduce the cancer mortality gap between England and similar countries, and narrow health inequalities.

**Table 1:** Net survival estimates by route, persons 36 months post diagnosis (%), England 2012–2016.

Disease	Total EP route (2006–2016)	% EP of all cancers diagnosed	3Y survival: EP route (taken for 2012–2016)	3Y survival (highest possible)	50% rerouting of EP would result in incremental 3Y survival of (#) over 10 years
Breast	19,734	4	42.3	100 (screening)	5693
Lung	141,667	36	6.2	31.5 (outpatient)	17,920
Prostate	34,626	8	44.9	95.9 (GP referral)	8829
Bowel	89,818	24	35	92.5 (screening)	25,822

One approach to reducing EPs is to adopt “Re-routing”. Re-routing is used to denote identifying potential avoidable EP instances and tracing back to what action could have been taken earlier in the journey in the context of future patients with a similar profile. In some instances, EP is not avoidable. For example, in case of first seizure or heavy gastrointestinal bleeding without prior warning features.

### Re-routing EP as a means to addressing mortality

The overall incidence of cancer in England is the first layer of prioritisation. Though there are more than 200 types of cancer, just four types—breast, prostate, lung and bowel—together account for more than half (53%) of all new cases in the UK (2016–2018) [21] and it is expected that incidence and mortality contribution will continue to be dominated by these four in the coming years [22]. Within these four cancers, sub-types of progressive disease (where the patient would benefit from an earlier diagnosis), may be prioritised.

Next, we use the RTD dataset and note the difference in three year survival in England between the EP route and the route offering highest survival for these four disease types. We also note the % of patients presenting in the EP route in the same period. These point to the potential of re-routing and the possible patient survival benefits in doing so (Table 1) [20].

Laudicella et al. [23] concluded that reducing EP via increases in primary care referral could be an achievable target with large benefits to patients against modest additional costs. Research has shown improved patient outcomes for increasing referral for all cancers but less impact for colorectal cancer [4].

### Comparators indicate achievability

Another indicator of the potential to re-route patients presenting in the EP route is a simple comparison between the highest performing and lowest performing sub-regions. The lowest figure from a subregion might be a practical short-term target to set for England. For example, Wessex had a total EP route presentation (all cancers) of 17% in 2016 versus England overall at 19% [20]. The difference seems to be in higher levels of GP referral and USC categories leading to a lower EP% in general—this points to increasing GP and USC routes as a potential strategy to reduce EP.

### Reducing inequalities in cancer screening

The best performing area could be a practical benchmark to improve screening uptake and interventions intensified in the sub-regions/communities with the lowest uptake. Areas with similar deprivation scores show different screening uptakes [24] indicating potential to intervene. Efforts to increase invitations and conversions should be intensified with a particular focus on reducing inequalities in certain groups, due to deprivation, ethnicity, poor mental health and disabilities. Many different mechanisms for improving screening participation have been evaluated, ranging from interventions that target non-participants with additional invitations and reminders, to interventions aimed at informing and educating the general population, prior to screening invitation [25, 26].

### Actionable moments at a prior consult

**Fig. 1:**
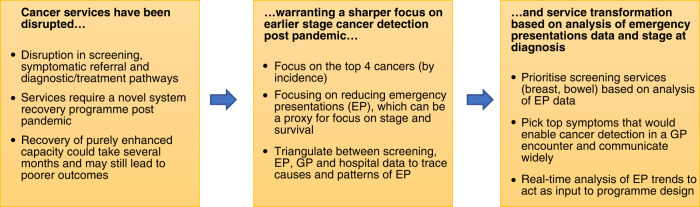
Prioritisation of and transformation of cancer services.

Understanding prior GP consultation data of EP patients [27] shows that while two-thirds of EP patients have had at least one prior consultation, about 1 in 5 had had 3 or more consultations with possible cancer-related symptoms. The health system therefore had access to some of these patients before they present as EP—this is a possible opportunity for an early intervention. Another study [28] of a sample of EP cancer patients concluded that approximately 19% of these represented missed opportunities at the primary care level. Conversely, both studies showed approximately one in three EP patients had no primary care/GP activity, and thus these could be potential populations to target for improving primary care access and earlier symptomatic presentation. Using linked data across primary and secondary care offers the opportunity for identification of actionable moments in near real time which could lead to earlier consideration of cancer diagnostic testing [Fig. 1].

## Potential opportunities for earlier testing and referral

**Fig. 2:**
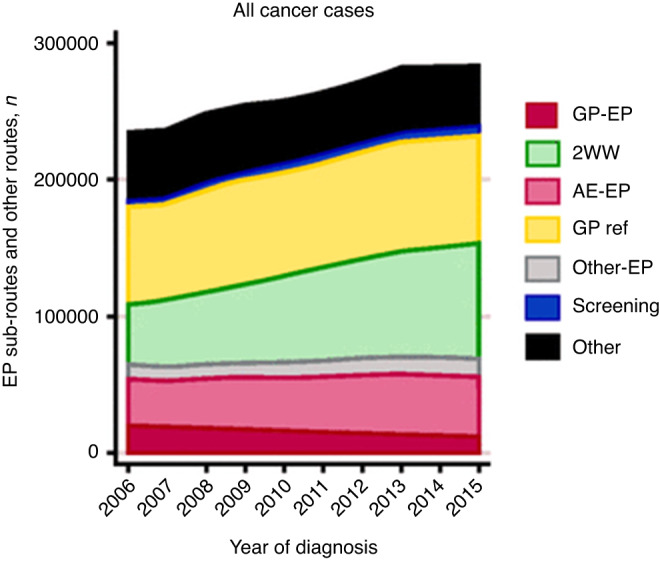
Reduction in cases presenting as emergency presentations (EPs)—2006–2015 (figure reproduced from Herbert et al. [45] with permission of authors and journal).

The cancer detection rate via USC referral pathways has continued to increase from 41% in 2009/10 to over 54% in 2021/22, while the USC conversion rate (predictive value of referral) for cancer diagnosis has dropped from 10% to 6% [3]. While primary care referral activity has increased, the proportion of patients diagnosed following the EP route has gradually reduced over the period 2006–2016, i.e. a total reduction from 24% to 19% diagnosed following EP over 10 years. A corresponding increase can be seen in cases diagnosed following USC (or 2WW) pathways (Fig. 2, [20]). However, approximately half of those with cancer present with vague non-specific symptoms which do not easily fit into a USC pathway [29]. These vaguer symptoms have a lower predictive value for cancer and patients can experience diagnostic.

### The proportion of cancers diagnosed following an EP

#### A data-driven approach

The aim is to identify which EPs are potentially avoidable through earlier diagnosis or screening. The primary question is—could this patient have been suspected of being at risk or having cancer earlier either in a community setting (screening), primary care setting (in a prior GP consultation) or a secondary care setting (in a hospital) with missed opportunities for earlier testing and detection?

Triangulation between EP and healthcare records could help us pinpoint the potential actionable moment to prioritise, based on the most commonly occurring patterns. This approach is still at an early stage and has mainly been done using retrospective data. A retrospective triangulation [30], linking primary care data from the National Cancer Diagnosis Audit to national cancer registration and RTD records demonstrated that patients presenting with non-specific vague symptoms experienced longer time intervals before diagnosis, were more likely to be diagnosed via an emergency and at a later stage of disease. This represents an opportunity for roll out and utilisation of rapid diagnostic centres (RDCs) offering increased diagnostic access including for CT for patients with non-specific symptoms such as weight loss.

### Data linkage and clinical decision support systems (CDSS)

With the development of electronic patient records and data linkage for example between primary and secondary care and cancer registries this offers the potential for near real time data and feedback to clinicians although significant barriers remain, including data linkage governance, time and cost implications.

There is evidence for CDSS to aid in consideration of important potential diagnoses such as cancer [31, 32]. Though barriers remain to their use and implementation including in routine clinical workflow such as alerts fatigue, inflexibility and lack of training [33], there is ongoing work into developing these systems to aid clinical decision making, though they are not as yet adopted in routine clinical practice.

Data at organisational level is available showing significant variation for example between GP practice, and secondary care levels and these can be used to inform quality improvement and improve patient outcomes [34, 35].

### Novel approaches to cancer detection

During the pandemic health services have gone through rapid changes including expansion of telephone, online and text access. This has posed both opportunities and challenges for patients and healthcare providers, with the potential for increased access; however, there is a concern of exacerbating pre-existing inequalities.

The potential for real-time text messaging to patients with integration into their electronic healthcare records has potential applications of direct relevance to earlier cancer detection. Including text message prompts to attend missed screenings [36], targeted messages for symptomatic presentation and as a consultation and safety netting tool [37]. These are areas which have been under-researched and offer potential for further studies and implementation.

Targeted encouragement of primary care consultations for possible cancer symptoms is an area for focus particularly in socioeconomically deprived areas with poorer cancer outcomes. An RCT [38] of targeted encouragement of GP consultations for possible cancer symptoms in at risk groups showed the feasibility of this approach.

Further development of CDSS integrating with existing electronic health records (EHRs) and newer digital and text message tools offer potential opportunities for prompting both patients and healthcare practitioners on earlier testing for cancer. This could include the integration of risk factors, clinical features and both existing (for example, blood tests) and novel tests to help aid in earlier detection.

With stretched healthcare workers including primary care as gatekeepers, could patients potentially be referred via their local pharmacy or even self-refer for initial triage testing? There is evidence for improved lung cancer outcomes following a patient awareness campaign and self-referral for chest x-ray (CXR) for patients aged 50 years and over with respiratory symptoms lasting 3 weeks or longer [39]. With clear evidence-based criteria, liberalising patient access via local pharmacies or via self referral to triage tests like CXR for respiratory symptoms and faecal immunochemical test (FIT) for bowel symptoms should be evaluated and encouraged if cost effective at a primary care and population level.

Given healthcare staff shortages up-skilling of expanded primary care health professionals such as advanced nurse practitioners, pharmacists, physician associates and social prescribers in recognition of potential cancer symptoms should be encouraged [40].

To meet the demands of earlier cancer diagnosis there is a need to both expand diagnostic capacity and use existing capacity better [41]. This includes expanding primary care direct access to tests, e.g. CT scanning, including via community RDCs [42].

Finally, the advent of novel tests such as multi-cancer early detection analysing circulating tumour cell-free DNA offers tantalising possibilities for earlier cancer detection both in screening [43] and symptomatic populations. While the data from large-scale clinical studies are awaited, it is worth considering some of the opportunities and challenges these novel tests pose and the frameworks for evaluation and translating into practice [44].

## Conclusion

In this paper, we have outlined the pandemic context on cancer services and suggest an alternative approach to cancer service transformation. With the significant challenge of an ageing population and increasing cancer incidence, an approach without strategic optimisation does not appear to be feasible especially in the context of an unprecedented backlog and workforce and diagnostic capacity constraints.

In order to implement this approach, it is recommended that further analysis is undertaken across EP-primary care-hospital EHR databases. Near real-time linkage of these three datasets aided by potential CDSS could ensure a more responsive strategy. Separate analyses are needed to quantify the geographic and ethnic disparities in USC referral and EP rates for certain cancers.

Based on the findings of data triangulation, interventions could include revised protocols (age of entry, risk stratified screening), choice of technology (such as CDSS), training and awareness, e.g. prioritised early symptoms or signs for both patients and healthcare professionals, with a particular focus on patient groups with later stage diagnoses to reduce health inequalities. This will inform the novel approaches outlined focusing on patient, healthcare professional/worker and system levels to aid in earlier cancer detection.

## Data Availability

Not applicable.
